# Follow-up and transition practices in esophageal atresia: a review of European Reference Network on rare Inherited and Congenital Anomalies (ERNICA) centres and affiliates

**DOI:** 10.1007/s00383-024-05865-z

**Published:** 2024-11-09

**Authors:** Natalie Durkin, Marco Pellegrini, Ramon Gorter, Graham Slater, Kate M. K. Cross, Benno Ure, Rene Wijnen, Frédéric Gottrand, Simon Eaton, Paolo De Coppi, ERNICA

**Affiliations:** 1 Stem Cell and Regenerative Medicine Section, Developmental Biology and Cancer Research, Zayed Centre for Research into Rare Disease in Children, Great Ormond Street Institute of Child Health, University College London, London, UK; 2 Department of Pediatric Surgery, Emma Children’s Hospital Amsterdam UMC, University of Amsterdam, Meibergdreef 9, Amsterdam, The Netherlands; 3 EAT—Esophegeal Atresia Global Support Groups e.V., Stuttgart, Germany; 4 Neonatal and Paediatric Surgery, Great Ormond Street Hospital for Children, London, UK; 5 Department of Pediatric Surgery, Hannover Medical School, Hannover, Germany; 6 Department of Pediatric Surgery and Intensive Care, Erasmus MC Sophia Children’s Hospital, Rotterdam, Netherlands; 7 INFINITE U1286, University of Lille, CRACMO Reference Centre for Rare Oesophageal Diseases, CHU Lille, Lille, France; 8 Research Area of Fetal, Neonatal, and Cardiological Sciences, Bambino Gesù Children Hospital, IRCSS, Rome, Italy

**Keywords:** Esophageal atresia, Transition, Follow-up, European reference networks

## Abstract

**Purpose:**

The purpose of this study was to understand the provision and distribution of esophageal atresia (EA) follow-up (FU) and transition services across European Reference Network for rare Inherited and Congenital Anomalies (ERNICA) member and affiliate centers.

**Methods:**

A REDCap questionnaire was sent to clinical leads of 18 ERNICA members and 14 affiliate centers.

**Results:**

29 of 32 centers responded (91%), the majority of which were highly specialized. Two-thirds had a dedicated EA clinic with a specialist multi-disciplinary team (MDT), offered to selected/complex patients only in 40% of centers. ERNICA centers were more likely to offer an MDT FU clinic than affiliates, with lack of resources most cited as a barrier to uptake (67%). Delivery of routine investigations was heterogeneous, particularly provision of three endoscopies over the course of FU (24%). Only 55% had a dedicated transition pathway, more prevalent in ERNICA centers (81% vs. 30%; *p* < 0.01). Self-reported awareness of ERNICA and European Society for Pediatric Gastroenterology Hepatology and Nutrition (ESPGHAN) guidance for FU and transition was poor (28%).

**Conclusion:**

Despite the existence of European follow-up and transition guidelines, their delivery is not uniform and may be limited by lack of awareness of the guidelines and a lack of resources.

**Supplementary Information:**

The online version contains supplementary material available at 10.1007/s00383-024-05865-z.

## Introduction

Esophageal atresia (EA) is associated with respiratory, nutritional, and gastrointestinal sequelae requiring surveillance into adulthood. Children and adults with EA suffer from long-term, multi-system morbidity secondary to both the underlying pathology itself and the surgical correction, which have been shown to significantly affect quality of life [1–3]. A recent meta-analysis reviewing over 800 patients > 11 years old with an average of 27 years post-EA repair identified high prevalence of gastrointestinal (42.4% GERD, 57.8% dysphagia, 12.4% metaplasia), respiratory (33.3%), neurological (11.7%), and musculoskeletal sequelae. The prevalence of patients defined as underweight was also high (19.6%), with 1.2% necessitating ongoing nutritional support [4]. These results are consistent with those of a previous meta-analysis prior to 2014, highlighting the need for active management and surveillance of EA patients into adulthood [5].

European Reference Network for rare Inherited and Congenital Anomalies (ERNICA) members form a network of expert European centers, together with associated patient support groups, that aim to reduce health inequalities and improve care; affiliate partner centers extend this collaborative network geographically. Evidence-based consensus statements composed by both ERNICA and the European Society for Pediatric Gastroenterology Hepatology and Nutrition (ESPGHAN) together with the North American Society For Pediatric Gastroenterology, Hepatology & Nutrition (NASPGHAN) aim to provide uniform EA FU protocols resulting in optimized patient care [6, 7]

During consensus statement development, a 100% consensus was reached that EA patients should be managed in specialized centers, defined as those with a minimum average caseload of five new EA cases per year. Incidentally, this is also the requirement for ERNICA recognition as an expert centre for EA, as opposed to status as an affiliate partner. Clear consensus (100%) was also reached on the need for multi-disciplinary follow-up, including an established transition process. Although both the published literature and the ERNICA and ESPGHAN–NASPGHAN guidelines make a clear case, follow-up and transition practices of EA patients across Europe remain largely unknown and patient organizations have reported concerns about variability of delivery [8]. The purpose of this study was to understand the provision and distribution of EA follow-up and transition services across ERNICA member and affiliate centers, to identify awareness and implementation of existing guidance across these centers, and to act as a benchmark to understand how services evolve over time.

## Methods

A questionnaire of follow-up and transition practices in EA, designed with and endorsed by members of ERNICA and EAT, was sent via REDCap to ERNICA-registered departmental clinical leads between December 2020 and January 2021 (Supplementary Appendix 1). The survey was divided into five domains which broadly correlated with themes of ERNICA and ESPGHAN–NASPGHAN guidance of EA follow-up: information about the center, provision of follow-up services, routine investigation and access to transition services, and proton-pump inhibitor use. At the time of survey, the questionnaire was sent to 19 registered ERNICA centres and 16 affiliate partner centers; three were subsequently excluded as adult-only centers (1 ERNICA, 2 affiliates). Centers were sent two reminder emails 1 week apart prior to being individually contacted for a response.

Data were compiled and analyzed using GraphPad Prism (v10.2). Continuous variables are described as median (range). Categorical variables are described as percentages, with statistical tests performed using Fisher’s exact or Chi-squared, with a *p* < 0.05 considered statistically significant.

## Results

**Fig. 1:**
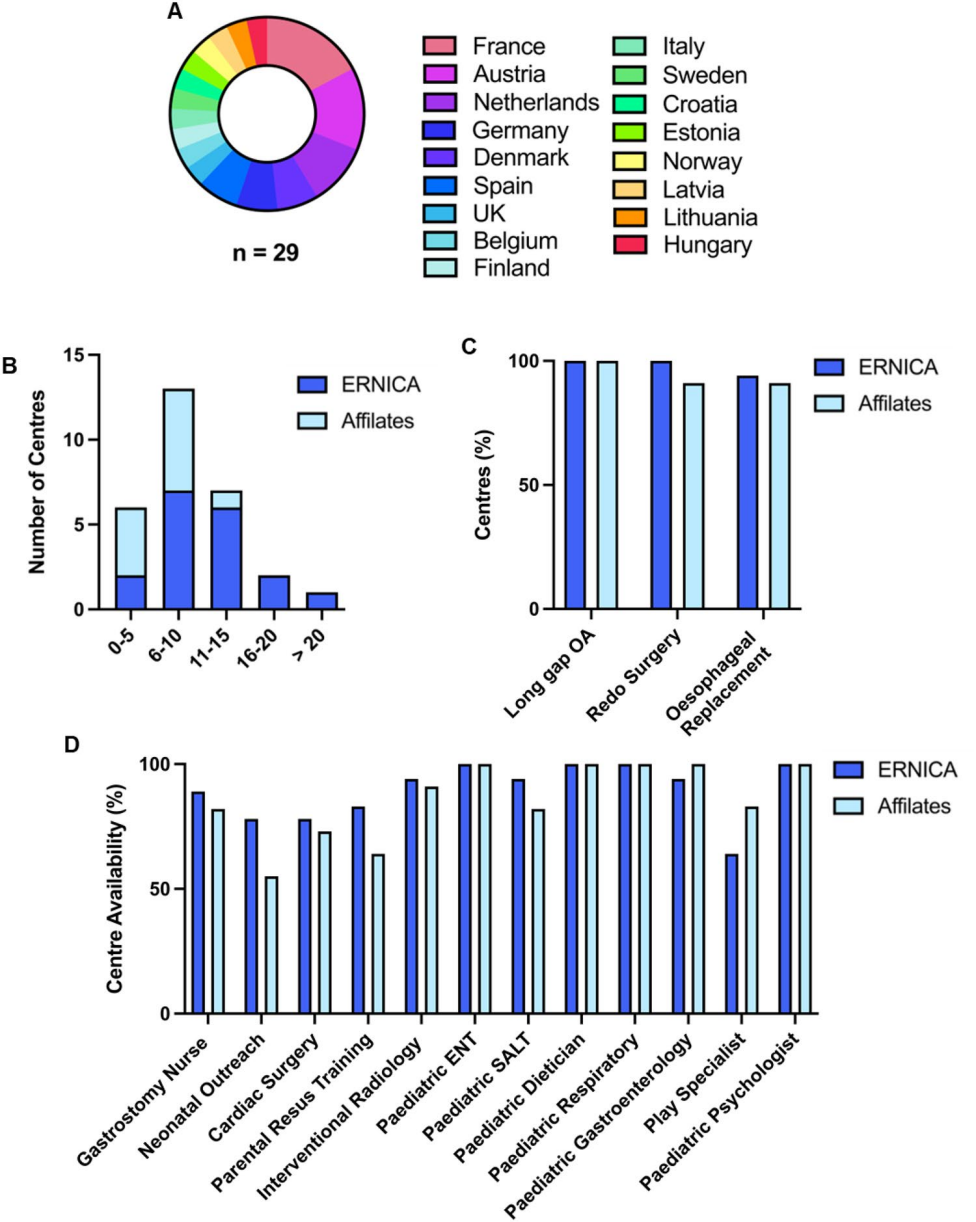
ERNICA and affiliate centres have comparable caseloads **A** 29 of 32 centres (91%) responded from 17 countries in Europe. **B** 79% of all centres manage > 5 new EA cases/year. ERNICA centres were more likely to have > 10 cases/year than affiliate centres. **C** The majority of centres were highly specialized managing long-gap EA and performing re-do and replacement surgery. **D** All centres offered a broad range of EA-related health services

The response rate was high; of the 32 pediatric ERNICA/affiliate centers, 29 responded (91%, 18/18 ERNICA, 11/14 affiliate), representing 17 European countries (Fig. 1A). The majority of centers were highly specialized; all reported managing long-gap patients, 97% reported performing esophageal re-do surgery, and 93% offering replacement surgery (67% gastric, 22% colonic, 11% jejunal) (Fig. 1C). 79% reported > 5 new EA cases/year (Fig. 1B), with ERNICA centers more likely to have > 10 new cases/year than affiliate centers (50 vs. 9%; *p* = 0.043). All centers offered a broad range of allied EA-related services, with access to pediatric gastroenterology, otolaryngology, pulmonology (respiratory), dieticians, and psychology in almost 100%. Most centers also offered neonatal outreach (69%), resuscitation training (76%), gastrostomy specialist nurses (86%), play specialists (76%) with access to cardiac surgery (76%), and interventional radiology (93%) services, although provision of these was less uniform (Fig. 1D).

**Fig. 2:**
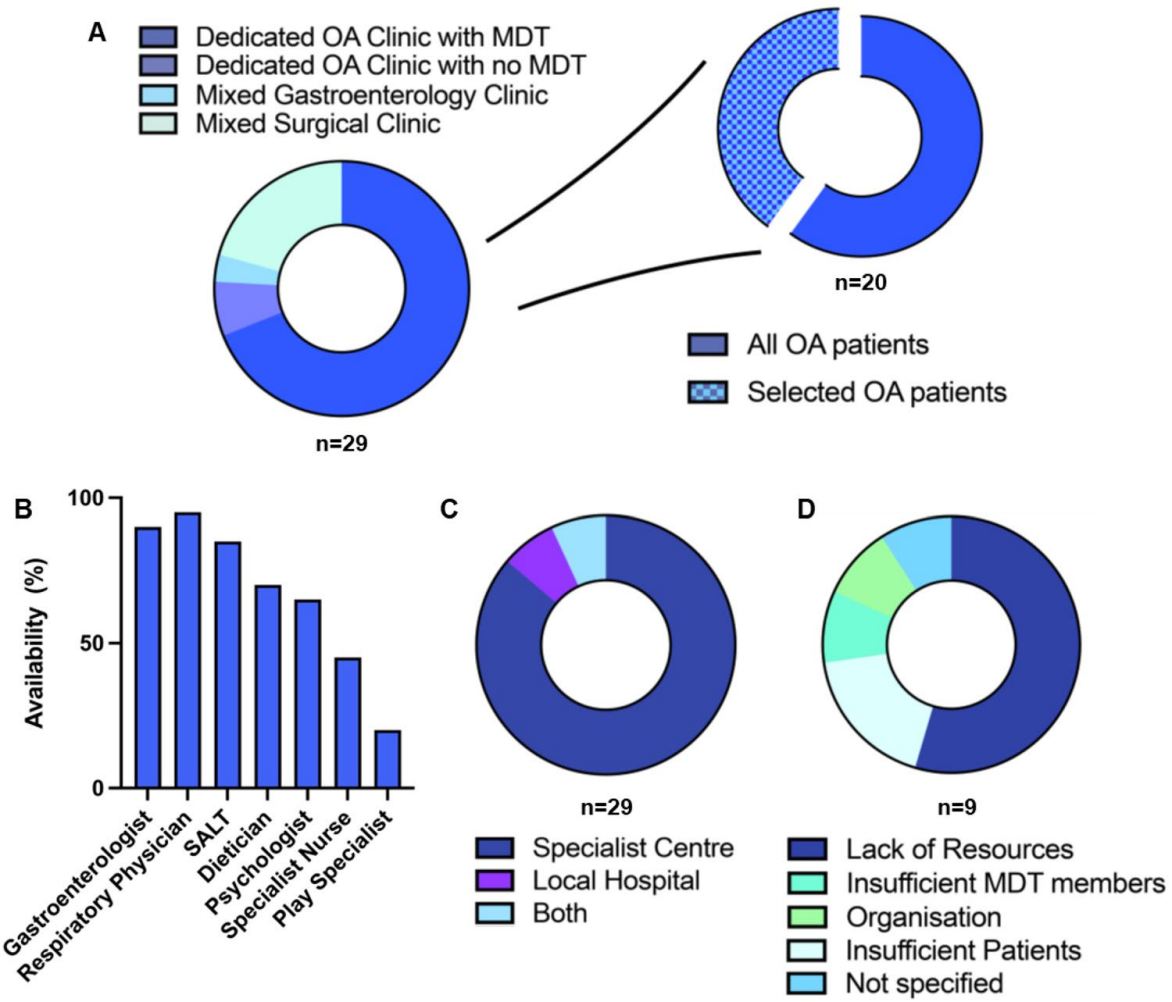
ERNICA and affiliate centres offer similar follow-up services **A**. Two-thirds of centers had a dedicated EA clinic with a specialist MDT. All EA patients attended specialist MDT clinics in 60% of centers, while 40% of centers offered MDT clinics only to selected complex EA patients. **B**. A broad range of services were available where MDT clinic was provided. **C**. Follow-up was predominantly offered at specialist hospitals, with limited availability at local services. **D**. Lack of resource was the most cited reason for lack of provision of MDT clinics

Two-thirds of centers had a dedicated EA clinic with a specialist multi-disciplinary team (MDT), accessible by all EA patients in 60% of centers, but selected/complex patients only in 40% (Fig. 2A). Where no MDT clinic existed, EA patients were followed up in routine surgical clinics (17%), gastroenterology clinics (3%), or a dedicated EA clinic with a single specialist but no MDT (7%). ERNICA centers appeared more likely to offer an MDT FU clinic than affiliates (78 vs 55%), although this was non-significant. Specialist EA MDT clinics were led by a consultant surgeon in 90%, and gastroenterology in 10%. Additional members of the MDT consisted of speech and language therapists (85%), dieticians (70%), psychologists (65%), gastroenterologists (90%), pulmonologists (95%), play specialists (20%), and specialist nurses (45%) (Fig. 2B). MDT clinics had been established for > 5 years in most centers with their reported presence (80%). Where MDT clinics were not yet available (*n* = 9), 67% reported plans to adopt this in the next 5 years, with a lack of resources the most cited reason for lack of uptake (67%, *n* = 6), insufficient patients (22%, *n* = 2), and insufficient members of MDT (11%, *n* = 1, Fig. 2D). Follow-up was predominantly offered at specialist hospitals, with limited availability at local hospitals to the patient (14%, Fig. 2C)). Of note, 97% reported access to psychology services, independent of whether this was in an MDT clinic; however, only 52% reported assessment with a validated quality of life tool during follow-up, with no difference observed between ERNICA and affiliate centers (50 vs 55%; *p* = ns).

**Fig. 3:**
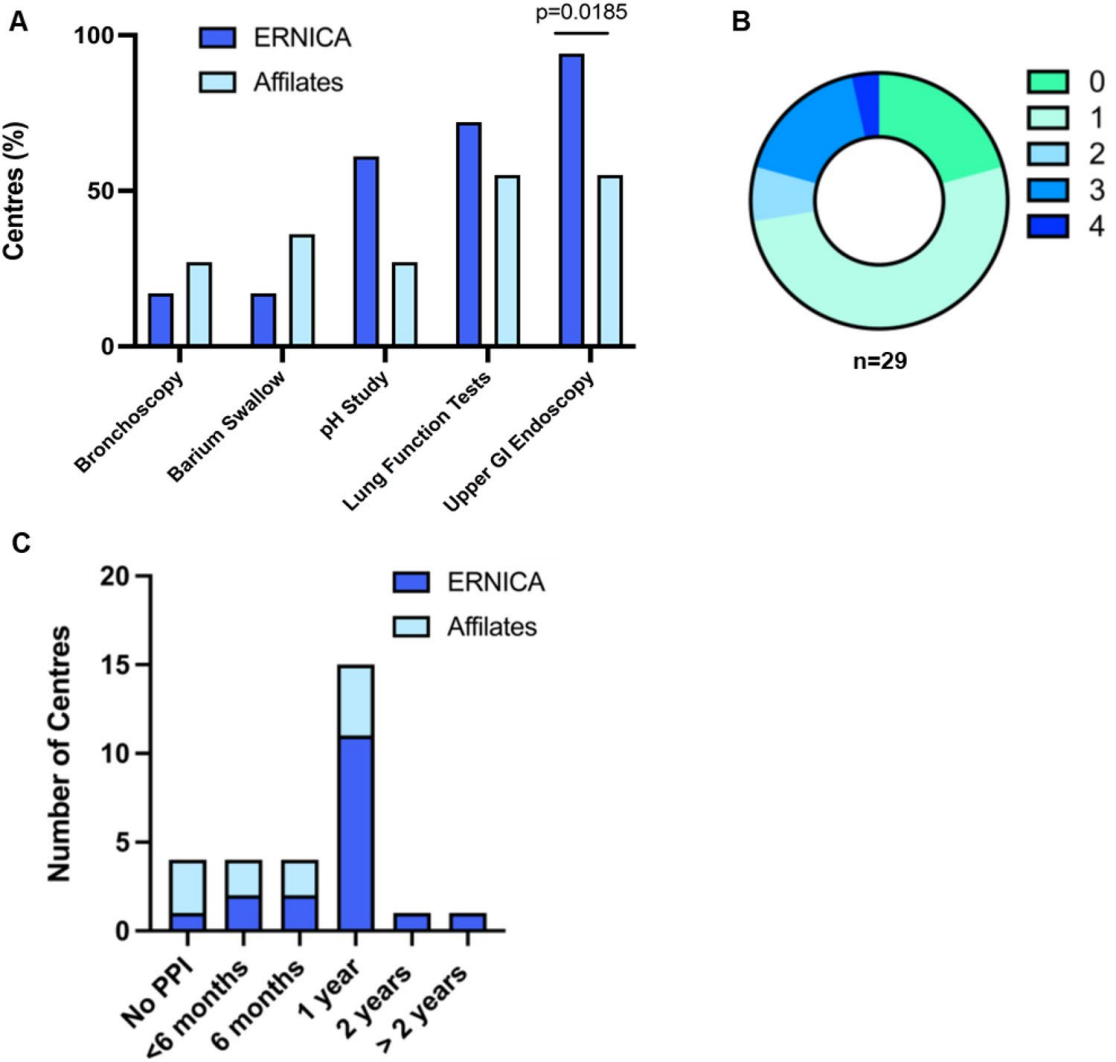
Delivery of routine investigations in asymptomatic EA patients is heterogeneous across ERNICA and affiliate centres **A**. Routine delivery of pH/impedance study, lung function tests, and endoscopy varied, although uptake was broadly higher in ERNICA centres. **B**. The ERNICA-ESPGHAN guidance of three endoscopies across the course of childhood FU was adhered to by only 24% of centres. **C**. 86% of all centres routinely prescribed Proton-Pump Inhibitor (PPI), although length of therapy was not standardised

Delivery of routine investigations in asymptomatic EA patients was heterogeneous. Of all investigations, surveillance endoscopy was the most frequently offered (79%), with first endoscopy at a median of 1 year (4 months–14 years). ERNICA centers were more likely to offer at least one endoscopy during FU (94% vs. 55%; *p* = 0.01), but delivery of three endoscopies over the course of FU as per ERNICA and ESPGHAN–NASPGHAN guidance was universally poor (24%) (Fig. 3A, B). Lung function tests were reported to be routinely performed at least once in 62% of centers at a median of 5 (1–15) years, more frequently offered in ERNICA than affiliate centers (72 vs. 45%; *p* = ns). 48% of centers offered a surveillance pH impedance study at a median of 1 year (1–5 years), again with a higher but non-significant uptake in ERNICA centers (61 vs. 27% *p* = 0.07). Routine use of bronchoscopy and barium swallow, not recommended by the ERNICA guidelines, were offered in 21% and 24% centers, respectively, more frequently routinely performed in affiliate than ERNICA centers (27 vs 17% and 36% vs. 17%; *p* = ns) at a median of 1 year (1 month–5 years).

Uptake of prophylactic anti-acid treatment was high, with 86% of centers reporting routine prescription of Proton-Pump Inhibitor (PPI) post-EA repair, although length of therapy was not standardized (1 year (< 6 months- > 2 years) (Fig. 3C). At discontinuation of therapy, 55% routinely performed pH/impedance studies, 16% barium swallow, and 32% performed no investigation.

**Fig. 4:**
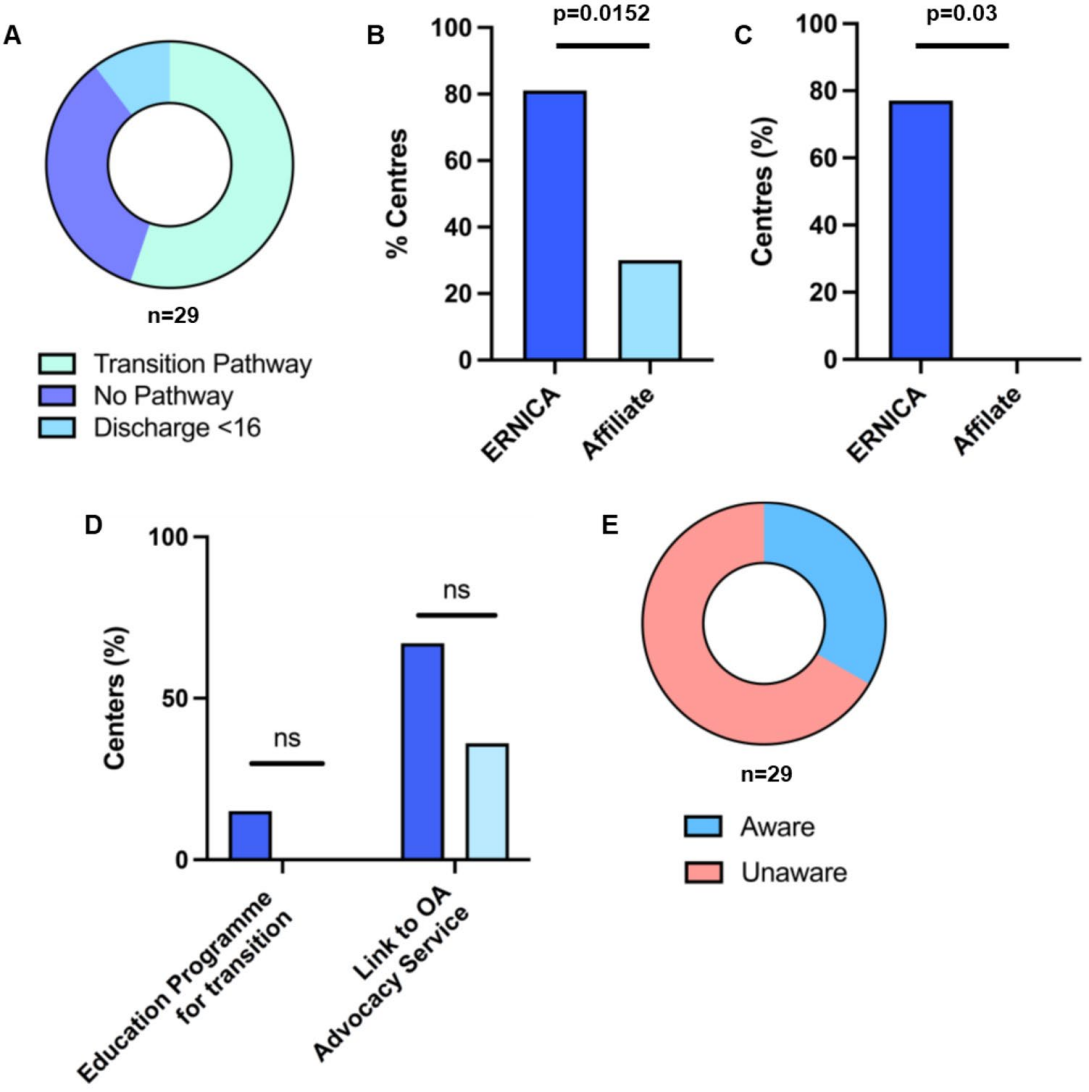
Transition appears to be more established amongst ERNICA centres **A** 55% of centers had a dedicated transition pathway for EA patients. The existence of a transition pathway (**B**) and a dedicated transition clinic with an adult specialist with an interest in EA (**C**) were higher in ERNICA centers. **D** A specific education program for transitioning EA adolescents existed in only two centers, with poor routine signposting to links to nationally recognized patient charities. **E** Self-reported awareness of ERNICA and ESPGHAN–NASPGHAN guidance for FU and transition of EA patients was universally poor

Only 55% of all centers reported the presence of a transition pathway (Fig. 4A), entered at a median of 18 (16–19) years, although an education program for transitioning EA adolescents only existed in two centers (7%). ERNICA centers were more likely to have a transition pathway than affiliates (81 vs. 30%; *p* = 0.0152) (Fig. 4B). Transition programs included a dedicated EA transition clinic with adult specialists with self-declared interest in EA in 63%, to either adult gastroenterology (65%), or general surgery (35%). ERNICA centers were far more likely to provide a specific transition clinic with adult specialists than affiliate centers (77% vs. 0%; *p* = 0.03) (Fig. 4C). Three centers report discharging EA patients from pediatric surgical follow-up prior to 16 years (10%) to adult general surgeons or pediatric gastroenterology. Only 41% of centers reported routinely offering a link to a nationally recognized EA patient charity (Fig. 4D). Overall, self-reported awareness of international guidance for FU and transition in EA was universally poor (28%) (Fig. 4E), with slightly higher awareness in ERNICA centers than affiliates (33 vs 18%; *p* = ns).

## Discussion

ERNICA is a network of expert multi-disciplinary healthcare professionals from specialist centers across Europe, partnered with patient advocacy and support groups who provide invaluable input from a patient perspective. Its main aim is to pool disease-specific expertise, knowledge, and resources on congenital anomalies to reduce health inequalities by standardizing practices, facilitating research and disseminating high-quality information. To that end, ERNICA consensus statements for the ‘Follow-Up and Framework’ of EA were formulated in 2018 in conjunction with EA patient organizations, based on the highest grade of evidence available [6]. Pre-existing ESPGHAN–NASPGHAN guidance for evaluation and treatment of gastrointestinal and nutritional complications in EA children were taken into consideration during consensus development, with ERNICA guidance covering more surgical aspects of follow-up (e.g., consideration of indication for fundoplication, treatment of recalcitrant strictures, etc.) compared with the mainly gastroenterological perspective of the ESPGHAN–NASPGHAN guidance [7].

Although ERNICA centers self-reported a higher volume of new EA cases per annum, provision of highly specialized surgical services (e.g., re-do/replacement surgery) as well as excellent availability of EA-associated medical and allied specialties were offered by both ERNICA and affiliate centers. Despite this, only two-thirds used these resources within an MDT clinic setting for EA patients, which was often only utilized for complex patients alone, rather than available to all. Whilst complex cases (e.g., recurrent fistula, re-do surgery, recalcitrant strictures, or long-gap EA) clearly require multi-disciplinary input [9], the high presence of associated pathologies, such as dysphasia, GERD, nutritional difficulties, and respiratory problems, which are often asymptomatic and may go unrecognized, indicate why speech and language therapy, gastroenterology, dietary, and pulmonology input and assessment as a minimum should be offered to all patients. In addition to the clinical need, it is also what EA patients and their families have asked for; focus groups have highlighted that patients and their parents want standardized MDT follow-up programmed during both child and adulthood under the guidance of one coordinating physician, to better understand all the aspects and consequences of EA. This expertise was felt to be lacking in general practitioners/primary care physicians [10, 11]. This study highlighted relative homogeneity in the self-reported responsible clinician across European centers, with 90% being pediatric surgeons.

The provision of routine investigations in asymptomatic EA patients was surprisingly heterogeneous and varied widely from that of both the ERNICA and ESPGHAN–NASPGHAN guidance; both recommend a pH/impedance study, LFTs, and a total of three endoscopies over the course of childhood follow-up. Of note, neither endorse barium swallow or bronchoscopy as a matter of routine. On the whole, ERNICA centers appeared to follow guidance more closely than affiliate centers, although the delivery of three endoscopies during childhood follow-up was universally poor, followed by only 24% of centers. With histological evidence of Barrett’s oesophagus found to be as high as 43% in the adolescent EA population [12], this approach should be strongly encouraged. Interestingly, on a survey of individual expert agreement with ESPGHAN guidance sent to ESPGHAN, NASPGHAN, and EUPSA members, 91% of those surveyed agreed in principle with the provision of three endoscopies, although from our survey, this does not appear to be currently offered in centers coordinating EA follow-up, highlighting a discordance between what expert clinicians think and what is actually being done [13]. Both ERNICA and ESPGHAN–NASPGHAN guidances also recommend the routine prescription of PPIs for all patients post-operatively, with the provision of a pH/impedance study at discontinuation of therapy. Whilst uptake of PPI prescription was widely followed post-EA-repair (86%), this was discontinued without a pH/impedance study, the gold standard for GERD diagnosis, in nearly half the centers. These results were consistent with that found in the INoEA survey, where there was high agreement that PPIs should be prescribed post-operatively but low agreement on the use of pH manometry on discontinuation [13].

Overall, just over half the centres provided formal transition pathways, with relative uniformity of implementation, with the majority reporting handover to adult gastroenterologists at 18 years of age. Transition was more established within ERNICA centers, which were more likely to offer dedicated transition clinics with adult specialists and adolescent education pathways. Our results correlate with the published experience of the self-reported provision of transitional care within congenital colorectal diseases, which was found to be present in only 44% of centers interviewed [14]. It also correlates with the documented current adult EA experience; several studies have highlighted a paucity of adult EA care, with one international study of 1100 adult patients documenting that 50% had no follow-up [3, 4]. With high rates of gastrointestinal and respiratory morbidity and increasing reports of esophageal carcinoma within the adult EA cohort [15, 16], the clinical need for this is unquestionable. However, the holistic benefit of transition for the patient and family should also not be underestimated. A large qualitative meta-synthesis of adolescent transition experiences on transfer from pediatric to adolescent care describes feelings of not belonging and being redundant to the process [17]. This was echoed in an EA-specific cross-sectional qualitative survey of patients and parents undergoing transition; five themes of feelings of abandonment, a cultural shift, a shift in responsibility, living with uncertainty, and a lack of any transition process were described [18]. The need for young adults to be valued as collaborators in their transfer to adult care is crucial, especially as the general transition between adolescence and adulthood is frequently associated with the desire to become independent from parents, caregivers, and those in perceived positions of authority as rapidly as possible. There are multiple examples of how structured transition pathways and education programs to guide adolescents through the transition process improve patient knowledge and improve outcomes, both in EA specifically, and other chronic pediatric conditions, such as inflammatory bowel disease, cystic fibrosis, asthma, and diabetes [19, 20].

As the ERNICA consensus statements and ESPGHAN–NASPGHAN guidelines are not standards, they are at the discretion of the clinician or unit to follow; however, most clinicians would not argue with the need for MDT follow-up and formalized transition care. What, then, are the barriers to achieving these more widely for all EA patients? The surprisingly low awareness of the consensus statements, although slightly higher in ERNICA centers themselves, may partially explain the variability in their uptake. A lack of resources, as alluded to by centers where MDT clinics did not exist, may also contribute to difficulty in provision of the recommendations from consensus statements and guidelines, although it was encouraging to note that the majority had plans to establish MDT clinics within the next five years. It may be that these recommendations are unattainable for centers with less accessibility to specialist investigations, and affiliate centers should be included in the future guideline revision to reflect this and offer alternative solutions, including clear criteria of when to refer patients to expert centres for specialist investigations and/or treatment. Finally, the existence guidelines with differing recommendations for the routine follow-up of the asymptomatic EA child (e.g., regarding PPIs for prevention of reflux) may not help clinicians determine which is the best framework to follow. Recent publication of the comprehensive INoEA guidance on transition and adult follow-up of EA patients now provides a specific and unified approach to follow for transition of EA patients into adulthood [21]. ERNICA and ESPGHAN are now working more closely together and hopefully future revisions of the ERNICA Consensus Statements and ESPGHAN–NASPGHAN Guidelines can be more closely aligned, or even amalgamated and endorsed by other relevant bodies, including INoEA, patient organizations, and national pediatric surgical and gastroenterological associations. Clear recommendations endorsed by multiple bodies will hopefully empower clinical teams to obtain the resources required for effective follow-up and transition clinics.

This initial survey has limitations—all surveys are limited by a tension between obtaining information and overburdening respondents, and often interpretation is limited by the desire to know additional information. For example, it would have been extremely useful to know what criteria are used in each center to accept patients into MDT clinics. Nevertheless, the survey provides useful information that can be taken forward to improve the knowledge of the consensus statements in expert and affiliate centers, and also to aid future development of unified guidelines across the different organizations to improve the transition of EA patients across Europe and beyond.

## Conclusions

ERNICA centers and affiliate partners have similar caseloads and availability of specialist EA services. The majority of centers offer specialist MDT clinics and dedicated EA transition pathways, but some inequality of services between ERNICA and affiliate centers exists. Where no MDT clinic existed, two-thirds of centers are planning to establish this within the next 5 years. Despite the existence of European follow-up and transition guidelines, their delivery is not uniform and may be limited by lack of awareness of the guidelines and a lack of resources. More work is required to make these guidelines more accessible and useable by centers to ultimately improve the standard of care for all EA patients.

## Supplementary Information

Below is the link to the electronic supplementary material.

Supplementary file1 (PDF 50 KB)

## Data Availability

Available from the corresponding autor on reasonable request.
