# Supplementary material for: Follow-up and transition practices in esophageal atresia: a review of European Reference Network on rare Inherited and Congenital Anomalies (ERNICA) centres and affiliates
Source: Pediatr Surg Int. 2024 Nov 9;40(1):300. doi: 10.1007/s00383-024-05865-z (PMC11550284; doi:10.1007/s00383-024-05865-z)
Supplement: Supplementary file 1 — Supplementary file1 (PDF 50 KB) [file 383_2024_5865_MOESM1_ESM.pdf]

Follow Up And Transition In Esophageal Atresia

Dear all,We would be very grateful if you could fill in the following questionnaire about the care of patients with esophageal atresia +/- tracheo-esophageal fistula (EA+/-TEF) on behalf of your centre as the clinical lead.The aim is to understand current follow-up and transition practices in Europe. This has been developed at the request of the TOFS (UK EA Patient support group, a member of EAT) Medical Advisory Board and has been fully endorsed by TOFS, and approved by Professor Benno Ure and Professor Frederic Gottrand, leads of the ERNICA Esophageal Diseases Workstream.It takes less than 10 minutes. For any questions, please email n.durkin@ucl.ac.uk.Thank you very much in advance.

Your centre

Which centre do you currently work at?	◯ Erasmus University Medical Centre Rotterdam ◯ Radboud University Hospital Nijmegen ◯ Utrecht University Medical Center ◯ Amsterdam UMC ◯ GOSH- Great Ormond Street Hospital for Children ◯ Universitatsmedizin Mannheim ◯ Hannover Medical University ◯ University Hospital Leuven ◯ Helsinki Children's Hospital ◯ AOP Padua ◯ Karolinska University Hospital, Stockholm ◯ Centre Hospitalier Regional Universitaire de Lille ◯ CHU Paris - Hopital Necker-Enfants Malades ◯ CHU Paris - Hopital Robert Debre ◯ Hopital Beaujon ◯ CHU de Marseille - Hopital de la Timone ◯ GHU Paris-Sud - Hopital Antoine Beclere ◯ Rigshospitalet Copenhagen ◯ Odense University Hospital ◯ Oslo University Hospital ◯ University Hospital Graz ◯ Kepler University Hospital Linz ◯ Sozial Medical Center East, Wien ◯ General Hospital of Vienna /Medical University of Vienna ◯ University Hospital Centre Zagreb ◯ Tartu University Hospital, Estonia ◯ Children's Clinical University Hospital, Riga ◯ Hospital of Lithuanian University of Health Sciences Kauno Klinikos ◯ Vilnius University Santaros Clinics ◯ Hospital Clinic of Barcelona ◯ La Paz, Madrid ◯ Hospital de Sant Joan de Deu, Barcelona ◯ Vall D'Hebron University Hospital, Barcelona ◯ Clinical institute for medical genetics, University medical centre, Ljubijana ◯ Semmelweis University, Institute of Genomic Medicine and Rare Disorders

For all the following questions, please answer with reference to universal practice in your clinical centre.

Approximately how many new EA +/- TEF cases do you have per year at your centre?	◯ 0-5<br>◯ 6-10<br>◯ 11-15<br>◯ 16-20<br>◯ More than 20<br>◯ Don't know

Complex cases

Do you manage long gap/type A/B (Gross classification) EA in your centre?	◯ Yes<br>◯ No
Do you perform redo EA surgery in your centre?	◯ Yes<br>◯ No
Do you perform esophageal replacement surgery in your centre?	◯ Yes<br>◯ No
In the case of replacement, which substitute does your centre prefer?	◯ gastric transposition<br>◯ colonic transposition<br>◯ jejunal transposition<br>◯ other

Which of the following associated services and allied health professionals do you have at your centre (select all that apply)?

☐ Gastrostomy nurse specialist ☐ Neonatal outreach nurse specialist ☐ Cardiac surgery
☐ Parental resuscitation training ☐ Paediatric interventional Radiology ☐ Paediatric ENT Surgeon
☐ Paediatric Speech and Language Therapy ☐ Paediatric Dietician ☐ Paediatric Respiratory Physician
☐ Paediatric Gastroenterology ☐ Paediatric Play Specialist ☐ Paediatric Psychologist

Access to follow-up services

Is standard follow up of EA/TEF cases in your centre:	◯ Mixed outpatient clinic with consultant surgeon only<br>◯ Mixed outpatient clinic with paediatric gastroenterologist only<br>◯ Dedicated clinic for EA/TEF patients only with single consultant surgeon OR paediatric gastroenterologist but no other multidisciplinary team members<br>◯ Dedicated EA/TEF clinic with specialist multidisciplinary team?
Is the specialist multi-disciplinary team clinic for all EA +/- TEF patients or only for selected patients dependent on clinical need?	◯ All patients<br>◯ Selected patients
Which specialities are present at the EA +/- TEF multidisciplinary team? Please tick all that apply.	☐ Speech and language therapist<br>☐ Dietician<br>☐ Psychologist<br>☐ Gastroenterologist<br>☐ Respiratory physician<br>☐ Play specialist<br>☐ Specialist nurse
Which speciality leads the specialist multidisciplinary team clinic?	◯ Paediatric Surgeon<br>◯ Paediatric Gastroenterologist<br>◯ General Paediatrician<br>◯ Paediatric respiratory
How long has this clinic been established?	◯ < 12 months<br>◯ 12-24 months<br>◯ 2-5 years<br>◯ > 5 years
What are the reasons for not having a multidisciplinary team clinic?	☐ Lack of resource<br>☐ Insufficient patients<br>☐ Insufficient members of MDT available<br>☐ Other
Other reason (please state)	__________________________________
Are you keen to establish a specialist EA +/- TEF multidisciplinary team clinic in the next 5 years?	◯ Yes<br>◯ No
Is your follow up of EA +/- TEF patients in a specialist centre or at a local hospital?	◯ Specialist centre<br>◯ Local hospital<br>◯ Both
Do you offer a quality of life assessment using a validated instrument at some point during the course of FU in EA children?	◯ Yes<br>◯ No
Do EA children have access to specialist counselling or psychology services?	◯ Yes<br>◯ No

Routine Investigation

Are antacid medications routinely prescribed for all patients after correction of EA?	◯ Yes<br>◯ No
Which medication do you primarily prescribe?	◯ Proton pump inhibitor<br>◯ H2 antagonist
How long do you routinely prescribe this medication for?	☐ < 6 months<br>☐ 6 months<br>☐ 1 year<br>☐ 2 year<br>☐ >2 years
Regardless of indication for antacid therapy (reflux or prophylaxis), at discontinuation of this, which of the following do you routinely perform?	☐ 24 hour pH study<br>☐ pH impedance study<br>☐ Barium swallow<br>☐ None of the above

For EA patients, regardless of symptoms, which of the following do you routinely perform during the course of follow up?

Lung function tests?	◯ Yes<br>◯ No
At which age / ages (years)?	__________________________
Endoscopy?	◯ Yes<br>◯ No
At which age / ages (years)?	__________________________
pH study?	◯ Yes<br>◯ No
At which age / ages (years)?	__________________________
Barium_swallow?	◯ Yes<br>◯ No
At which age / ages (years)?	__________________________
Bronchoscopy?	◯ Yes<br>◯ No
At which age / ages (years)?	__________________________

Transition

Do you discharge patients with EA/TEF from specialist surgical or gastroenterology FU prior to 16 years old?	◯ Yes<br>◯ No
Who do you discharge EA +/- TEF patients to?	☐ GP<br>☐ Adult general surgeon<br>☐ Local paediatrician<br>☐ Paediatric gastroenterologist<br>☐ Paediatric respiratory physician
Do you have a specific transition process or pathway for all EA +/- TEF patients?	◯ Yes<br>◯ No
At which age do you transition EA +/- TEF patients?	◯ 12-15<br>◯ 16<br>◯ 17<br>◯ 18<br>◯ >18
Do you routinely transition to an adult specialist with a defined interest in adult EA?	◯ Yes<br>◯ No
Who do you transition EA +/- TEF patients to?	◯ Adult gastroenterologist<br>◯ Adult surgeon
Who do you transition EA +/- TEF patients to?	◯ General Practitioner<br>◯ Adult gastroenterologist<br>◯ Adult general surgeon<br>◯ Other
Do you have a joint EA +/- TEF clinic with an adult speciality?	◯ Yes<br>◯ No
Do you have a specific education programme for transitioning EA +/- TEF adolescents?	◯ Yes<br>◯ No
Are you aware of any existing EA +/- TEF follow up or transition guidelines? If so, which?	______________________________
Do you routinely offer a link with a nationally recognised EA +/- TEF patient advocacy service?	◯ Yes<br>◯ No
Please feel free to provide comments here.	______________________________
